# Psychosocial Functionality and Predictors in Bariatric Surgery Candidates

**DOI:** 10.31083/AP47647

**Published:** 2025-10-24

**Authors:** Rahime Gök, Ezgi Şişman, Elif Tatlıdil, Aslıhan Polat

**Affiliations:** ^1^Department of Psychiatry, Kocaeli University Faculty of Medicine, 41001 Kocaeli, Türkiye; ^2^Psychiatry Clinic, Tekirdağ Yaşam Hospital, 41001 Tekirdağ, Türkiye; ^3^Psychiatry Clinic, Kocaeli City Hospital, 41001 Kocaeli, Türkiye; ^4^Community Mental Health Center, Kocaeli University, 41001 Kocaeli, Türkiye

**Keywords:** obesity, bariatric surgery, psychosocial functioning, depression, depressive symptoms, eating disorders, preoperative care

## Abstract

**Background:**

Obesity is a critical global health issue with increasing prevalence. Although bariatric surgery is effective, relapses are common. Pre-bariatric functioning may significantly influence these relapses.

**Objective:**

To evaluate psychosocial functioning in individuals undergoing bariatric surgery, examining depressive symptoms, self-esteem, body satisfaction, disordered eating symptoms, and sociodemographic factors. This cross-sectional study identifies predictors of psychosocial functioning to guide interventions for sustained postoperative well-being.

**Methods:**

The study included 175 individuals (81.7% female) attending routine preoperative evaluations at Kocaeli University Faculty of Medicine. Most participants (94.3%) were morbidly obese (body mass index (BMI) ≥40). Psychosocial functioning was assessed using the Obesity-Related Problems Scale (OP-S), with 51.4% scoring in the severe range (≥60). Depressive symptoms (Beck Depression Inventory (BDI)), Rosenberg Self-Esteem Scale (RSES), body satisfaction Scale (BSS), and Eating Disorder Examination Questionnaire (EDE-Q) were also evaluated. Correlation and regression analyses identified predictors of psychosocial functioning.

**Results:**

The mean OP-S score was 55.81 ± 24.77. OP-S scores were significantly correlated with depressive symptoms (r = 0.462, *p* = 0.001), disordered eating symptoms (r = 0.410, *p* = 0.002), self-esteem (r = –0.322, *p* = 0.004), and body satisfaction (r = –0.240, *p* = 0.018). Regression analysis identified depressive symptoms (β = 0.24, *p* = 0.02) and disordered eating symptoms (β = 0.20, *p* = 0.03) as significant predictors.

**Conclusion:**

Depressive symptoms and disordered eating symptoms are predictors of psychosocial functioning among individuals undergoing bariatric surgery. Addressing these factors through psychiatric evaluations can enhance psychosocial functioning, reduce relapse risk, and improve quality of life. Multidisciplinary care is essential in bariatric treatment.

## Main Points

• Depressive symptoms are the strongest predictor of severe psychosocial impairment in bariatric surgery candidates.

• Eating disorder symptoms independently contribute to psychosocial distress, but their predictive value is lower than depressive symptoms.

• Body dissatisfaction, not gender, is a key factor in preoperative psychosocial functioning.

• More than half of bariatric surgery candidates experience severe psychosocial distress, highlighting the need for comprehensive psychological assessment.

• Preoperative screening for depressive symptoms and disordered eating could help identify high-risk patients and improve surgical outcomes.

## 1. Introduction

Obesity is a chronic, progressive, and recurrent condition characterized by an excessive or abnormal accumulation of body fat that negatively impacts both physical health and social well-being. It is widely recognized as a significant global health issue due to its steadily increasing prevalence and its strong association with numerous chronic diseases and complications, which impose a substantial burden on healthcare systems worldwide [1]. Obesity is a chronic, progressive, and recurrent condition characterized by an excessive or abnormal accumulation of body fat that negatively impacts both physical health and social well-being. The global rise in obesity has led to growing concern, particularly due to its connection with serious health conditions and its impact on healthcare systems [2].

Most people affected by obesity try various strategies such as diet, exercise, or behavioral support to lose weight. However, while these strategies may lead to initial weight reduction, maintaining the achieved weight loss over an extended period presents a significant difficulty, with many individuals experiencing weight regain despite continued efforts to adhere to prescribed regimens [3, 4]. Because traditional methods don’t always work for everyone, bariatric surgery has become a widely accepted alternative for those with more severe obesity. Still, surgery is not a guaranteed fix—many patients regain weight or face ongoing challenges [5]. This potential for weight regain is a shared concern among both patients and clinicians. While several physiological and metabolic factors contribute to this issue, accumulating evidence suggests that psychiatric and psychosocial factors play a crucial role in determining postoperative outcomes [6]. In addition to the inherent risks associated with surgical procedures, including potential complications and the need for lifelong medical follow-up, the presence of psychiatric disorders or maladaptive psychological coping mechanisms may significantly impact an individual’s ability to adhere to the necessary lifestyle modifications required for long-term success after surgery. As a result, evaluating the psychological and psychosocial functioning of individuals who are candidates for bariatric surgery is essential in order to identify potential risk factors that may hinder successful postoperative weight maintenance and overall well-being [7].

A study has shown that individuals seeking bariatric surgery tend to have a higher prevalence of psychiatric comorbidities compared to severely obese individuals who opt not to undergo surgical intervention for weight loss [8]. This observation underscores the importance of conducting thorough psychiatric evaluations in individuals with obesity, particularly those who are considering bariatric surgery. Preoperative psychosocial dysfunction, adverse mental health conditions, and untreated psychiatric disorders have been identified as factors that may contribute to suboptimal postoperative weight loss outcomes and may even lead to increased psychological distress following surgery [9]. Given these findings, it has been emphasized that identifying patients who may benefit from more intensive preoperative psychiatric treatment, as well as closer postoperative psychiatric follow-up, is a crucial step in maximizing the long-term success of bariatric surgery [10]. There is growing evidence to suggest that implementing targeted psychotherapeutic interventions in the preoperative period, particularly for individuals with underlying eating disorders, may significantly enhance the long-term outcomes of bariatric surgery [11]. Addressing maladaptive eating behaviors, improving emotional regulation, and strengthening psychological resilience prior to surgery can help optimize treatment outcomes and reduce the likelihood of postoperative weight regain. In recognition of the unique psychiatric challenges associated with obesity and bariatric surgery, some researchers have even proposed the establishment of “bariatric psychiatry” as a distinct subspecialty within the field of psychiatry [12]. The increasing emphasis on psychiatric and psychological factors in the context of obesity treatment highlights the need for a multidisciplinary approach that integrates mental health support as a fundamental component of bariatric surgery programs.

A recent study suggest that ongoing psychological stress and internalized weight stigma can strongly influence how people with obesity function socially and emotionally. According to stigma theory, people who experience weight-related discrimination may begin to adopt those negative beliefs about themselves, which often leads to reduced self-esteem and difficulties in social interactions [13]. The transactional model of stress and coping offers a similar view, proposing that constant sources of stress—like dissatisfaction with one’s body or feeling judged—can make it harder to regulate emotions and adapt to challenges [14]. Supporting these ideas, earlier research has shown links between low self-esteem, poor body image, and both a lower quality of life and worse outcomes after bariatric surgery [15, 16]. These patterns highlight how important it is to consider self-esteem and body satisfaction when assessing a patient’s psychosocial readiness for surgery.

Psychiatric approaches to obesity treatment extend beyond weight regulation and encompass various aspects of mental health and psychosocial functioning. These approaches aim to address maladaptive eating behaviors, enhance social functioning, improve self-esteem and body satisfaction, and target psychiatric symptoms such as anxiety, depression, compulsive eating, and binge eating disorder [17]. Obesity has been consistently linked to significant reductions in quality of life, affecting not only physical health but also psychological and social well-being. Psychiatric interventions offer valuable support by addressing the emotional burden associated with obesity, such as mood disturbances, low self-esteem, and social withdrawal. Assessing quality of life in this context allows clinicians to evaluate treatment impact more holistically and tailor interventions to improve both psychological resilience and daily functioning.

Previous studies have consistently shown that bariatric surgery outcomes are influenced by various psychosocial factors, such as depressive symptoms and disordered eating [18, 19]. However, there is a notable lack of research specifically examining psychosocial functioning as a distinct construct in the preoperative period. Given that psychosocial functioning reflects the cumulative impact of psychological difficulties on daily life and social roles, and that it constitutes a core component of obesity-related quality of life [20], identifying its preoperative determinants may offer valuable insights for tailoring psychiatric evaluations and interventions prior to surgery. Such efforts may, in turn, contribute to better psychosocial adaptation and adherence in the postoperative period.

Therefore, the present study aims to comprehensively assess psychosocial functioning in individuals seeking bariatric surgery at a university hospital by examining key psychological variables, including depressive symptoms, self-esteem, body satisfaction, and disordered eating symptoms. Additionally, this study seeks to determine whether specific sociodemographic factors, body mass index (BMI), and mental health variables can serve as significant predictors of psychosocial functioning in this patient population. By identifying the most relevant psychological and psychosocial determinants of preoperative functioning, this research aims to inform psychiatric evaluations in bariatric surgery candidates and contribute to the development of targeted interventions that enhance long-term surgical success.

## 2. Methods

This study was designed as a cross-sectional observational study and included all individuals who were referred to the Health Board outpatient clinic for a routine preoperative psychiatric evaluation before undergoing bariatric surgery for morbid obesity. The study period spanned from January 2016 to December 2016, during which all eligible candidates meeting the inclusion criteria were invited to participate.

To be eligible for inclusion in the study, participants had to be between the ages of 18 and 65, ensuring that both younger and older patients within the adult age range were represented. Additionally, only individuals who were cognitively and educationally capable of understanding and completing the required assessments were included, ensuring the reliability and validity of self-reported measures. Exclusion criteria included individuals with severe cognitive impairment, active psychotic disorders, or current substance use disorders, as such conditions could compromise the psychiatric evaluation process or the accuracy of self-reported data. Those who declined to participate or later withdrew consent were also excluded. All patients were scheduled for bariatric surgery due to a clinical diagnosis of morbid obesity, and no further medical exclusion criteria were applied beyond the psychiatric criteria described above. This inclusive approach ensured that the study sample was representative of the broader population of patients undergoing bariatric surgery within the given timeframe.

Prior to participation, all individuals were thoroughly informed about the purpose, scope, and procedures of the study. The voluntary nature of participation was emphasized, and participants were given the opportunity to ask any questions before providing written informed consent. Ethical considerations were carefully adhered to throughout the study, with approval obtained from the Non-Interventional Clinical Research Ethics Committee of Kocaeli University. This ensured compliance with ethical guidelines and safeguarded participant rights, privacy, and confidentiality in accordance with established research ethics principles.

### 2.1 Data Collection Tools

*Sociodemographic form:* Participants provided information on age, education, marital status, employment, income, smoking and alcohol use, emotional eating and weight loss product use.

*Body Mass Index (BMI):* Height and weight were measured using a calibrated scale and stadiometer. BMI was calculated as weight (kg) divided by height squared (m^2^).

*Beck Depression Inventory (BDI):* This 21-item scale assesses depression severity, with higher scores indicating greater depressive symptoms. It has been validated in Turkish populations [21].

*Rosenberg Self-Esteem Scale (RSES):* The 10-item self-esteem subscale of the RSES was used to evaluate self-esteem levels [22, 23]. The Rosenberg Self-Esteem Scale (RSES) is a Likert-type self-assessment tool measuring self-esteem, with response options ranging from “strongly agree” to “strongly disagree”. While the full scale consists of 63 items across 12 subscales, the 10-item self-esteem subscale is commonly used in research [23]. Higher scores indicate greater self-esteem. This study utilized the 10-item self-esteem subscale.

*Body Satisfaction Scale (BSS):* The Body Satisfaction Scale (BSS), developed by Berscheid *et al*. (1973) [24], was adapted to Turkish by Gökdoğan (1988) [25]. BSS was adapted with cultural considerations, ensuring validity through expert review and reliability via test-retest (r = 0.88). It includes 25 items for females and 26 for males, rated on a 5-point Likert scale from “extremely satisfied” to “not satisfied at all”. The scale assesses overall body appearance, face, body parts, and torso, with higher scores indicating greater body satisfaction. This study used the total BSS score to measure body satisfaction.

*Obesity-Related Problems Scale (OP-S):* The Obesity-Related Problems Scale (OP-S) was developed based on the Health-Related Quality of Life (HRQoL) scale to assess the impact of psychosocial functioning on the quality of life in individuals with obesity [20]. The OP-S consists of 8 items that assess the extent to which individuals feel burdened by their weight in various social situations. Scores range from 8 to 32 and are standardized (0–100). Higher scores indicate greater psychosocial impairment, categorized as mild (<40), moderate (40–59), or severe (≥60). In this study, the total OP-S score was calculated and converted into the OP-S-S score using the formula. The Turkish version of the OP-S used in this study was based on a previously conducted validity and reliability study by Polat *et al*. [26] (2014). In this adaptation study, the scale demonstrated good internal consistency (Cronbach’s α = 0.82) and a two-factor structure (social and psychological). Item-total correlations were within acceptable limits, and construct validity was supported by significant associations with measures of depression, body image, and psychological well-being.

*Eating Disorder Examination Questionnaire (EDE-Q):* The EDE-Q, developed by Fairburn and Cooper in 1993, is a self-report scale. Its Turkish validity and reliability were established by Yucel *et al*. [27] in 2011. A score of 4 or higher on any subscale is considered indicative of an eating disorder. In this study, participants with a score of 4 or higher were considered at risk for a probable eating disorder based on screening criteria, consistent with previous validation study [28].

All psychometric instruments used in this study had previously undergone Turkish validation and have demonstrated acceptable to excellent internal consistency. Reported Cronbach’s alpha coefficients from Turkish validation studies are as follows: BDI, α = 0.80–0.85 [21]; RSES, α = 0.76 [22]; BSS, α = 0.86 [29]; Eating Disorder Examination Questionnaire (EDE-Q), α = 0.90 [27]; and OP-S, α = 0.84 [20]. These coefficients support the reliability of the instruments used in the current study.

### 2.2 Procedure

Participants completed self-report measures during their preoperative psychiatric evaluation. Of the 175 individuals approached, 11 declined to participate, and one withdrew after opting out of surgery. Data were analyzed using correlation and regression analyses to identify psychosocial predictors of preoperative functioning.

### 2.3 Statistical Analysis 

All statistical analyses were conducted using IBM SPSS Statistics for Windows, (Version 22.0; IBM Corp., Chicago, IL, USA). Descriptive statistics were used to summarize sociodemographic and clinical characteristics. Continuous variables are reported as means and standard deviations (SD), and categorical variables as frequencies and percentages. The assumption of normality was assessed for all continuous variables using the Shapiro–Wilk test. The results indicated that the distributions of key continuous measures, including the Obesity-Related Problems Scale standardized score (W = 0.983, *p* = 0.051) and Rosenberg Self-Esteem Scale (W = 0.987, *p* = 0.084), did not significantly deviate from normality. However, BDI scores significantly deviated from normality (W = 0.953, *p* < 0.001). However, BDI scores significantly deviated from normality (W = 0.953, *p* < 0.001). Despite this, parametric analyses were retained due to the sufficiently large sample size (n = 175) and the robustness of these methods to moderate deviations from normality. Given that the distribution of BDI scores deviated from normality, Spearman correlation was additionally performed as a non-parametric alternative. Furthermore, to assess the robustness of the Pearson correlation coefficients, a bootstrap analysis with 1000 samples was conducted. The results confirmed the reliability of the Pearson correlation between BDI and OP-S scores (mean r = 0.30, 95% CI: 0.19 to 0.41). Associations between OP-S subgroups and categorical sociodemographic and behavioral variables (e.g., gender, marital status, obesity classification) were examined using Pearson’s chi-square test. For the obesity classification variable, chi-square assumptions were violated (>20% of expected cell frequencies <5); therefore, Fisher’s exact test with Monte Carlo simulation (10,000 replications) was applied. For all other categorical comparisons, assumptions for Pearson’s chi-square test were met, with no expected frequencies below 1 and overall expected counts sufficient for valid interpretation given the total sample size (N = 175). The corresponding test statistics are reported in the Results section. Pearson correlation coefficients were calculated to explore bivariate associations among psychosocial functioning (OP-S standardized scores), depressive symptom severity, self-esteem, body satisfaction, and eating disorder symptoms. A multiple linear regression analysis was conducted to identify independent predictors of psychosocial functioning. Variables entered into the model included depressive symptom scores (BDI), self-esteem (RSES), body satisfaction (BSS), eating disorder symptoms (EDE-Q), BMI, and gender. A backward stepwise regression was also performed to assess the stability and parsimony of the model. All regression assumptions, including linearity, homoscedasticity, independence of residuals, and absence of multicollinearity, were examined. The level of significance was set at α = 0.05, and all *p*-values are reported to three decimal places. Analyses were two-tailed. To evaluate multicollinearity among predictors in the regression model, variance inflation factors (VIFs) were calculated. Furthermore, Harman’s single-factor test was performed to assess common method bias due to the use of self-report measures, using an unrotated principal component analysis.

## 3. Results

The participants’ mean age was 38.39 (±11.60), and the mean BMI was 47.50 (±7.07). Other sociodemographic data of the participants are presented in Table 1.

**Table 1.** **BMI and Sociodemographic data of the participants**.

		n	%
Body Mass Index			
	Obese (BMI: 30–39)	10	5.7
	Morbid obese (BMI ≥40)	165	94.3
Sex			
	Female	143	81.7
	Male	32	18.3
Marital status			
	Married	116	66.3
	Single	59	33.7
Educational status			
	Primary school	91	52.0
	High school	46	26.3
	College	38	21.7
Employment status			
	Employed	65	37.1
	Unemployed	110	62.9
Total monthly income			
	Low	29	16.6
	Lower middle	54	30.9
	Upper middle	68	38.9
	High	24	13.7
Number of the living children			
	None	61	34.9
	One	27	15.4
	Two	50	28.6
	Three	23	13.1
	Four and more	14	8.0
Weight loss product use			
	User	23	13.1
	Non user	152	86.9
Emotional Eating			
	Yes	121	69.1
	None	54	30.9
Smoking			
	Non smoker	74	42.3
	Ex smoker	49	28.0
	Smoker	52	29.7
Alcohol use			
	User	24	13.7
	Non user	151	86.3

BMI, body mass index.

Table 2 summarizes the participants’ scale scores. Based on the EDE-Q, 52 participants (29.7%) met the screening criteria for disordered eating symptoms, while 123 (70.3%) did not. The distribution of OP-S scores across subgroups is shown in Table 3.

**Table 2.** **Findings on Participants’ Scale Scores**.

		Mean ± sd	Range
BDI		17.40 ± 10.82	0–41
RSES		20.49 ± 5.00	3–30
OP-S	Total	21.39 ± 5.94	8–32
	Standard	55.81 ± 24.77	0–100
BSS		76.56 ± 13.69	34–112
EDE-Q		3.26 ± 1.19	0–5.81

BDI, Beck Depression Inventory; RSES, Rosenberg Self-Esteem Scale; OP-S, Obesity-Related Problems Scale; BSS, Body Satisfaction Scale; EDE-Q, Eating Disorder Examination Questionnaire.

**Table 3.** **Obesity Related Problems Scale Scores**.

	n	%
Mild (OP-S <40)	49	28
Moderate (OP-S = 40–59)	36	20.6
Severe (OP-S ≥60)	90	51.4

OP-S, Obesity Related Problems Scale standardized scores. Note: Although OP-S scores were grouped descriptively (mild, moderate, severe) for interpretation, all inferential analyses, including correlation and regression, used the continuous OP-S total score as the outcome variable.

No significant associations were found between OP-S subgroups and obesity classification, gender, marital status, income, employment, education, use of weight loss products or emotional eating (Table 4).

**Table 4.** **Associations between OP-S subgroups and sociodemographic variables**.

Variable		Low OP-S	Moderate OP-S	High OP-S	χ ^2^	df	*p*
		N (%), n = 49	N (%), n = 36	N (%), n = 90			
Obesity classification						2	0.212*
	Obese (30 ≤ BMI < 40)	4 (8.2%)	0 (0%)	6 (6.7%)			
	Morbid obese (BMI ≥40)	45 (91.8%)	36 (100%)	84 (93.3%)			
Gender					3.530	2	0.171
	Female	37 (75.5%)	29 (80.6%)	79 (87.8%)			
	Male	12 (24.5%)	7 (19.4%)	11 (12.2%)			
Marital status					0.809	2	0.667
	Single	14 (28.6%)	13 (36.1%)	32 (35.6%)			
	Married	35 (71.4%)	23 (63.9%)	58 (64.4%)			
Income					1.093	6	0.982
	Low	10 (20.4%)	5 (13.9%)	14 (15.6%)			
	Lower middle	13 (26.5%)	12 (33.3%)	29 (32.2%)			
	Upper middle	19 (38.8%)	14 (38.9%)	35 (38.9%)			
	High	7 (14.3%)	5 (13.9%)	12 (13.3%)			
Employment					1.437	2	0.487
	Employed	19 (38.8%)	16 (44.4%)	30 (33.3%)			
	Unemployed	30 (61.2%)	20 (55.6%)	60 (66.7%)			
Education level					0.115	4	0.998
	Primary school	25 (51.0%)	19 (54.3%)	47 (52.2%)			
	High school	13 (26.5%)	9 (25.7%)	24 (26.7%)			
	College	11 (22.4%)	7 (20.0%)	20 (22.2%)			
Use of weight loss products					0.055	2	0.973
	User	6 (12.2%)	5 (13.9%)	12 (13.3%)			
	Non user	43 (87.8%)	31 (86.1%)	78 (86.7%)			
Emotional eating					0.006	2	0.997
	Yes	34 (69.4%)	25 (69.4%)	62 (68.9%)			
	None	15 (30.6%)	11 (30.6%)	28 (31.1%)			

Note: Values are presented as n (%). Chi-square (χ^2^) tests were used. Degrees of freedom (df) are indicated. Statistical significance was set at *p* < 0.05. *Fisher’s exact chi-square with Monte Carlo simulation.

However, OP-S scores correlated significantly with BDI, RSES, BSS, and EDE-Q scores (Table 5). Higher levels of depressive symptoms and disordered eating symptoms, body dissatisfaction, and lower self-esteem were linked to poorer psychosocial functioning.

**Table 5.** **Correlation of OP-S, BDI, RSES, BSS, EDE-Q scores and BMI**.

	BDI	RSES	BSS	EDE-Q	BMI
OP-S	r = 0.462,	r = –0.322,	r = –0.240,	r = 0.410,	r = –0.009,
	*p* = 0.001	*p* = 0.004	*p* = 0.018	*p* = 0.002	*p* = 0.882
BDI	-	r = –0.491,	r = –0.298,	r = 0.671,	r = 0.003,
		*p* = 0.001	*p* = 0.011	*p* = 0.001	*p* = 0.977
RSES		-	r = 0.368,	r = –0.241,	r = 0.021,
			*p* = 0.001	*p* = 0.015	*p* = 0.817
BSS			-	r = –0.153,	r = –0.002,
				*p* = 0.047	*p* = 0.991
EDE-Q				-	r = –0.768,
					*p* = 0.001

Statistical test used: Pearson correlation coefficient. r values represent Pearson correlation coefficients with corresponding *p*-values, reported to three decimal places.

Significant correlations were observed between BDI and RSES, BSS, and EDE-Q scores, as well as between RSES and both BSS and EDE-Q. BMI was not significantly correlated with OP-S-S, BDI, RSES, or BSS (all *p* > 0.05). However, a significant negative correlation was observed between BMI and EDE-Q (r = –0.768, *p* = 0.001) (Table 5).

Pearson correlation identified significant associations between OP-S and multiple variables, including BDI, RSES, BSS, and EDE-Q, as shown in Table 5. To address the non-normal distribution of BDI scores (Shapiro–Wilk W = 0.953, *p* < 0.001), both Pearson and Spearman correlation analyses were conducted. The correlation between BDI and OP-S scores was significant in both analyses (Pearson r = 0.462, *p* = 0.001; Spearman ρ = 0.308, *p* < 0.001). Additionally, a bootstrap analysis with 1000 samples confirmed the robustness of the Pearson correlation (mean r = 0.30, 95% CI: 0.19 to 0.41). These findings indicate a consistent and meaningful association between depressive symptoms and obesity-related psychosocial problems.

To determine predictors of OP-S, a linear regression analysis was conducted with these four variables, along with gender and BMI for clinical relevance (Table 6). The linear regression analysis revealed a statistically significant positive relationship between the OP-S and both BDI and EDE-Q, while no significant relationship was observed between the OP-S and other variables. Among the predictors, depressive symptoms (β = 0.24) demonstrated the strongest standardized association with psychosocial functioning, followed by severity of disordered eating symptoms (β = 0.20). This indicates that depressive symptoms have a relatively greater effect on psychosocial impairment, even when differences in measurement scales are taken into account. The model was statistically significant (F(6, 156) = 8.952, *p* < 0.001) and explained 25.6% of the variance in psychosocial functioning (R^2^ = 0.256). Although the backward stepwise model yielded a more parsimonious solution, its explanatory power (R^2^ = 0.171) was inferior to that of the full model (R^2^ = 0.256). Thus, the full model was preferred based on both statistical performance and theoretical coherence. The VIF values for all predictors ranged from 1.03 to 1.72, indicating no evidence of multicollinearity. Harman’s single-factor test showed that the first unrotated factor accounted for 31.6% of the total variance, suggesting that common method bias was not a substantial concern. In conclusion, BDI and EDE-Q were identified as predictors of psychosocial functioning in this regression model.

**Table 6.** **Linear Regression Model for the predictors of OP-S scores**.

	B	SE	β	*p*
BDI	0.56	0.24	0.24	0.02
RSES	–0.58	0.41	–0.12	0.16
BSS	–0.16	0.14	–0.09	0.25
EDE-Q	10.78	5.06	0.20	0.03
BMI	0.03	0.24	0.009	0.89
Sex	–1.51	5.06	–0.02	0.77

F = 8.952, df = 6, *p* < 0.001, R^2^ = 0.256. B, unstandardized coefficient; SE, standard error; β, standardized coefficient.

## 4. Discussion

### 4.1 Evaluation of Psychosocial Functionality: Findings and Implications

The analysis of psychosocial functioning, as measured by the OP-S, revealed that the average score among participants indicated a moderate level of psychosocial impairment. This finding suggests that while individuals seeking bariatric surgery experience notable challenges related to their psychosocial well-being, the severity of impairment may vary significantly across different patients.

A previous study examining the psychosocial consequences of obesity and the impact of bariatric surgery on quality of life have reported inconsistent findings. Previous research has yielded results that align with the present study, demonstrating moderate impairments in psychosocial functioning and quality of life before surgery [30]. However, another study has indicated a more pronounced decline in psychosocial well-being, suggesting that individuals with severe obesity may experience greater psychosocial distress and functional impairments than observed in the current sample [31]. These discrepancies may stem from variations in quality of life measures and differences in assessed subdomains.

### 4.2 The Impact of Depressive Symptoms on Pre-bariatric Psychosocial Functionality 

The analysis of depressive symptoms among study participants, as measured by the Beck Depression Inventory (BDI), indicated that the average score fell within the range typically associated with mild depressive symptomatology based on established cut-off points for the scale. This finding aligns with previous research indicating that depressive symptoms are relatively common among bariatric surgery candidates, with mild symptoms being among the most frequently reported [32]. The consistency of our results with the existing literature further supports the view that elevated levels of depressive symptoms are frequently present among individuals undergoing evaluation for bariatric surgery. Given the high rates of psychiatric comorbidities observed in individuals with severe obesity, it is not surprising that depressive symptoms emerge as a key factor influencing psychosocial functioning in this population.

A statistically significant relationship was identified between the psychosocial functioning component of quality of life and depressive symptoms, suggesting that individuals experiencing greater psychological distress tend to report poorer psychosocial well-being. Additionally, regression analyses confirmed that depressive symptom is a significant predictor of psychosocial functioning, indicating that higher levels of depressive symptoms are associated with greater impairments in overall quality of life. These findings are consistent with previous studies emphasizing the crucial role of depressive symptoms in preoperative adjustment [18, 32]. Based on the standardized coefficients, depressive symptoms (β = 0.24) emerged as the strongest predictor of psychosocial functioning, having a slightly larger effect than disordered eating symptom severity (β = 0.20), even when accounting for the different measurement scales [31]. Previous research highlights that depression before surgery is associated with lower postoperative quality of life [33] and increases the risk of less weight loss, weight regain, and the need for reoperation [34, 35]. In line with these findings, our results show that individuals with higher levels of depressive symptoms are more vulnerable to psychological distress, which has been linked to poorer postoperative outcomes. Additionally, depressive symptoms may contribute to prolonged hospital stays after surgery [35]. These findings emphasize the importance of early identification and treatment of depression during the perioperative period.

The strong association between depressive symptoms and psychosocial functioning may reflect a bidirectional relationship. On one hand, symptoms such as anhedonia, fatigue, and social withdrawal can diminish patients’ capacity to participate in meaningful interpersonal and occupational roles, thereby lowering their quality of life [15, 33]. On the other hand, ongoing functional impairments—particularly those arising from obesity-related stigma or physical limitations—may foster feelings of hopelessness, reduced self-efficacy, and affective dysregulation, which in turn exacerbate depressive symptomatology [13, 14]. These dynamics underscore the need to consider depressive symptoms not merely as a risk factor, but also as an outcome of impaired psychosocial functioning.

### 4.3 The Impact of Disordered Eating Symptoms on Pre-bariatric Psychosocial Functionality

In our study, 52 participants (29.7%) were identified as having disordered eating sypmtoms based on EDE-Q results. While this rate is consistent with some previous findings, other studies have reported higher prevalence rates of disordered eating symptoms among bariatric surgery candidates [36, 37]. One possible explanation for the relatively lower rate observed in our study is the tendency of participants to present themselves in a more favorable light during the preoperative evaluation process. Given that approval from the health board is required for surgery, some individuals may underreport symptoms related to disordered eating behaviors in an effort to secure medical clearance. Conversely, studies reporting lower rates of eating disorders than ours have often focused exclusively on binge eating disorder, rather than assessing a broader spectrum of eating disorder pathology [38]. The variability in reported prevalence rates across different studies likely stems from methodological differences, including variations in diagnostic criteria, assessment tools, and sample characteristics. The absence of standardized and universally accepted screening protocols for disordered eating symptoms in bariatric surgery candidates further complicates direct comparisons across studies. Given the well-documented comorbidity between obesity and disordered eating symptoms, careful and comprehensive screening for disordered eating behaviors should be prioritized in preoperative evaluations to ensure that psychological factors influencing treatment outcomes are adequately addressed.

Our findings indicate that the presence of disordered eating symptoms is significant predictor of psychosocial functioning, particularly in relation to quality of life. Consistently, studies examining quality of life in individuals with obesity have reported a statistically significant negative impact of eating disorders on overall well-being [36]. In our study, the strong negative relationship observed between disordered eating symptoms and psychosocial functioning may be attributed to the specific nature of the psychosocial domain of quality of life assessments, which primarily captures the psychological and emotional consequences of obesity. This reinforces the importance of addressing disordered eating behaviors as part of a multidisciplinary approach to optimize psychosocial well-being and surgical outcomes in bariatric patients.

Disordered eating behaviors, particularly those characterized by emotional eating or binge episodes, may operate as maladaptive strategies for coping with negative emotions in bariatric populations [6]. These behaviors are frequently accompanied by feelings of shame and self-criticism, which can promote avoidance of social situations and impair psychosocial engagement [39]. Furthermore, internalized weight stigma may heighten vulnerability to both disordered eating and social isolation, creating a self-perpetuating cycle that undermines psychological well-being [13]. Addressing this cycle in preoperative interventions may be critical to improving long-term surgical and psychosocial outcomes.

### 4.4 The Impact of Self-esteem and Body Satisfaction on Pre-bariatric Psychosocial Functionality

Participants’ mean RSES scores aligned with some studies [15, 40] but were higher than those reported elsewhere [41, 42]. These variations may stem from differences in education, employment, expectations, stigma exposure, and medical or social factors.

Direct comparisons of body satisfaction are challenging due to variations in assessment tools. However, extensive research links obesity to body dissatisfaction [43]. While prior studies found significant negative correlations between self-esteem, body satisfaction, and quality of life [44, 45], our study did not identify them as predictors. This may be due to their complex interplay with depressive symptoms, where dissatisfaction lowers self-esteem, contributing to depressive symptoms, which emerged as the primary predictor in our regression model.

Although self-esteem was not a predictor in our study, literature suggests that enhancing self-esteem may improve surgical outcomes [16]. Research on obese women supports self-esteem and body satisfaction as predictors of psychosocial functioning [29]. While not identified as key factors in our analysis, their significant associations with psychosocial functioning underscore their relevance in treatment. A previously published study by Polat *et al*. [29] (2019), for example, also examined psychosocial functioning in individuals with obesity; however, it focused solely on voluntarily enrolled women in a non-surgical outpatient lifestyle program. In contrast, the present study includes both men and women undergoing mandatory psychiatric evaluation as part of a bariatric surgery protocol and integrates additional variables such as disordered eating symptoms. These differences in sample characteristics, clinical context, and analytical scope highlight the unique contribution of the current study. Addressing body image concerns, particularly through psychotherapy, may provide therapeutic benefits [39].

### 4.5 Gender and Sociodemographic Factors: Their Association With Psychosocial Functionality

In our sample, psychosocial functioning was not significantly associated with gender, education level, income, or BMI. Although some studies have linked female gender and higher education to increased psychosocial distress in obesity [15, 45, 46, 47], these associations were not observed in our data, possibly due to the overriding influence of psychological factors such as depressive symptoms and disordered eating.

### 4.6 The Impact of BMI on Pre-bariatric Psychosocial Functionality

In our study, no significant differences in psychosocial functioning were observed between obese and morbidly obese participants, suggesting that BMI alone may not be a primary determinant of preoperative psychosocial well-being. While some studies have reported greater declines in mental health-related quality of life in individuals with class II obesity compared to those with class III obesity [48], others have linked higher preoperative BMI and comorbid depression/anxiety to increased postoperative BMI [49]. These discrepancies highlight the complex and multifaceted nature of psychosocial functioning in individuals with obesity, which extends beyond BMI classification alone. The lack of a significant BMI-related effect in our study suggests that psychosocial functioning is likely shaped by a broader range of psychological and social factors. This underscores the importance of adopting a more holistic approach in preoperative assessments, considering not only BMI but also the psychosocial and emotional context in which obesity-related distress occurs.

### 4.7 What is the Significance of Assessing Psychosocial Functioning and Conducting Psychiatric Evaluations in Pre-bariatric Processes?

The rising prevalence of obesity as a global health concern and the difficulty in maintaining well-being after bariatric surgery highlight the critical need to address underlying psychosocial factors. Psychological predispositions that negatively affect quality of life are associated with suboptimal outcomes following bariatric surgery. Identifying these individuals during the preoperative phase or early postoperative period and providing targeted psychological interventions can significantly enhance both quality of life and surgical success [50]. Furthermore, evidence from other studies indicates that managing psychological distress before and after surgery leads to more favorable outcomes [51, 52]. In our study, Depressive symptom severity and disordered eating risk were identified as negative predictors of psychosocial functioning. Therefore, recognizing the potential impact of depressive symptoms and disordered eating symptoms on bariatric surgery outcomes and implementing psychiatric evaluation and interventions to improve psychosocial functioning are key to optimizing results.

### 4.8 Limitations

The sample was selected from patients attending medical faculty hospitals, and this population may differ from those seeking care at state or private hospitals. Additionally, participants may have tended to present themselves more favorably to obtain approval for bariatric surgery. The cross-sectional design of our study is another limitation, as it does not allow for generalizations or the establishment of causal relationships. Moreover, the artificially created physical and psychological effects of the study environment may have led participants to exhibit atypical reactions, a phenomenon known as the Hawthorne Effect [53]. Reporting bias may also have influenced our results, given that the participants were a group actively seeking treatment. Although the OP-S is a validated and widely used measure of psychosocial functioning in obesity, it primarily assesses weight-related social impairment and may not capture broader emotional or cognitive dimensions of psychological distress. Its self-report format may also introduce social desirability bias, particularly in preoperative settings where patients may underreport difficulties in an effort to secure surgical approval. These limitations should be considered when interpreting the findings. Furthermore, although depressive symptoms and disordered eating symptoms were modeled as predictors of psychosocial functioning, some conceptual overlap with the OP-S scale cannot be fully ruled out. While the OP-S primarily targets social and behavioral impairments associated with obesity, certain items may be indirectly influenced by affective symptoms. This potential overlap should be acknowledged as a limitation of the current model and considered when interpreting the findings.

## 5. Conclusion

This study highlights the crucial role of depressive symptoms and disordered eating symptoms as significant predictors of psychosocial functioning in individuals preparing for bariatric surgery. Our findings suggest that higher levels of depressive symptoms and the presence of disordered eating symptoms are strongly associated with impaired psychosocial well-being in the preoperative period. Recognizing these predictors during preoperative psychiatric evaluations is essential for identifying patients at risk of poorer psychological outcomes, allowing for early intervention strategies that may enhance their overall treatment experience.

While much of the existing research on bariatric surgery focuses on postoperative outcomes, our study provides valuable insights into the pre-bariatric phase, emphasizing the psychological factors that shape preoperative well-being and potentially influence long-term success. However, despite the recognized importance of psychosocial risk factors in shaping post-surgical adjustment, the specific role of psychosocial functioning as a predictor of postoperative outcomes remains underexplored. Future studies are needed to clarify whether preoperative psychosocial functioning can serve as a reliable indicator of long-term adaptation and weight maintenance.

For obesity treatment to be truly effective and centered on the patient, it’s important to include regular psychological evaluations before surgery. Involving mental health professionals as part of the care team can help address key emotional and behavioral challenges—such as depression, low self-esteem, body image concerns, and disordered eating—before they become barriers to success. Catching and working on these issues early can make it easier for patients to adjust after surgery, stick to necessary lifestyle changes, and improve their overall well-being. Understanding the psychological factors that affect surgery outcomes may help clinicians tailor treatment plans to individual needs, which could support better long-term results.

## Public Significance Statement

This study highlights the critical role of psychiatric evaluations in individuals undergoing bariatric surgery. Identifying and addressing depressive symptoms and disordered eating behaviors in the preoperative period can significantly enhance patients’ psychosocial functioning and quality of life, thereby improving overall treatment outcomes. By integrating routine psychiatric assessments into obesity management, healthcare providers can develop more effective, patient-centered treatment plans, ultimately supporting long-term well-being and sustained surgical success.

## Availability of Data and Materials

Data supporting the findings of this study are not publicly available due to privacy and ethical considerations. However, they may be accessed upon reasonable request from the corresponding author, subject to approval by the Kocaeli University Ethical Review Board.

## References

[1] Mallik R, Carpenter J, Zalin A (2023). Assessment of obesity. *Clinical Medicine (London, England)*.

[2] Blüher M (2019). Obesity: global epidemiology and pathogenesis. *Nature Reviews. Endocrinology*.

[3] Carvalho AD, Turato ER, Chaim EA, Magdaleno R (2014). Weight regain among women after metabolic and bariatric surgery: a qualitative study in Brazil. *Trends in Psychiatry and Psychotherapy*.

[4] Veronese N, Facchini S, Stubbs B, Luchini C, Solmi M, Manzato E (2017). Weight loss is associated with improvements in cognitive function among overweight and obese people: A systematic review and meta-analysis. *Neuroscience and Biobehavioral Reviews*.

[5] Yücel B, Akdemir A, Gürdal Küey A, Maner F, Vardar E (2013). *Yeme bozuklukları ve obezite-tanı ve tedavi kitabı. 1st edn*.

[6] Kalarchian MA, Marcus MD (2015). Psychosocial Interventions Pre and Post Bariatric Surgery. *European Eating Disorders Review: the Journal of the Eating Disorders Association*.

[7] Rodolico A, La Rosa VL, Romaniello C, Concerto C, Meo V, Saitta G (2024). Personality dimensions, depression, and eating behavior in individuals seeking bariatric surgery: a cluster analysis. *Frontiers in Nutrition*.

[8] Kalarchian MA, Marcus MD, Levine MD, Soulakova JN, Courcoulas AP, Wisinski MSC (2008). Relationship of psychiatric disorders to 6-month outcomes after gastric bypass. *Surgery for Obesity and Related Diseases: Official Journal of the American Society for Bariatric Surgery*.

[9] Sarwer DB, Allison KC, Wadden TA, Ashare R, Spitzer JC, McCuen-Wurst C (2019). Psychopathology, disordered eating, and impulsivity as predictors of outcomes of bariatric surgery. *Surgery for Obesity and Related Diseases: Official Journal of the American Society for Bariatric Surgery*.

[10] Hilgendorf W, Butler A, Timsina L, Choi J, Banerjee A, Selzer D (2018). A behavioral rating system predicts weight loss and quality of life after bariatric surgery. *Surgery for Obesity and Related Diseases: Official Journal of the American Society for Bariatric Surgery*.

[11] Campos Del Portillo R, Matía Martín P, Castro Alija MJ, Martínez Olmos MÁ, Gómez Candela C (2022). Prevention of eating disorders in obesity. *Nutricion Hospitalaria*.

[12] Troisi A (2022). Emergence of bariatric psychiatry as a new subspecialty. *World Journal of Psychiatry*.

[13] Puhl RM, Heuer CA (2010). Obesity stigma: important considerations for public health. *American Journal of Public Health*.

[14] Lazarus RS, Folkman S (1984). *Stress, appraisal, and coping*.

[15] Rosenberger PH, Henderson KE, Grilo CM (2006). Correlates of body image dissatisfaction in extremely obese female bariatric surgery candidates. *Obesity Surgery*.

[16] Felske AN, Williamson TM, Scurrey SRM, Telfer JA, Campbell TS, Rash JA (2021). The Influence of Weight-Related Self-Esteem and Symptoms of Depression on Shape and Weight Concerns and Weight-Loss 12 Months After Bariatric Surgery. *Obesity Surgery*.

[17] Beck JS (2012). *The Beck Diet Solution: Train your brain to think like a thin person*.

[18] Sarwer DB, Wadden TA, Fabricatore AN (2005). Psychosocial and behavioral aspects of bariatric surgery. *Obesity Research*.

[19] Marek RJ, Williams GA, Mohun SH, Heinberg LJ (2017). Surgery type and psychosocial factors contribute to poorer weight loss outcomes in persons with a body mass index greater than 60 kg/m2. Surgery for Obesity and Related Diseases. *Surgery for Obesity and Related Diseases: Official Journal of the American Society for Bariatric Surgery*.

[20] Karlsson J, Taft C, Sjöström L, Torgerson JS, Sullivan M (2003). Psychosocial functioning in the obese before and after weight reduction: construct validity and responsiveness of the Obesity-related Problems scale. *International Journal of Obesity and Related Metabolic Disorders: Journal of the International Association for the Study of Obesity*.

[21] Hisli Sahin N (1989). Use of the Beck Depression Inventory with Turkish University Students: Reliability, validity and Factor Analysis. *Türk Psikoloji Dergisi*.

[22] Çuhadaroğlu F (1986). *Adolesanlarda benlik saygısı (Self-esteem in adolescents) [Unpublished medical speciality thesis]*.

[23] Rosenberg M (1965). *Society and the adolescent self-image*.

[24] Berscheid E, Walster E, Bohrnstedt G (1973). The happy American body: A survey report. *Psychology Today*.

[25] Gökdoğan F (1988). *Orta öğretime devam eden ergenlerde beden imajından hoşnut olma düzeyi*.

[26] Polat A, Turan H, Erzincan E, Tural Ü (2014). Turkish validity and reliability study of the Obesity-Related Problems Scale in obese individuals [Poster presentation]. *In 50th National Psychiatry Congress*.

[27] Yucel B, Polat A, Ikiz T, Dusgor BP, Elif Yavuz A, Sertel Berk O (2011). The Turkish version of the eating disorder examination questionnaire: reliability and validity in adolescents. *European Eating Disorders Review: the Journal of the Eating Disorders Association*.

[28] Mond JM, Hay PJ, Rodgers B, Owen C, Beumont PJV (2004). Validity of the Eating Disorder Examination Questionnaire (EDE-Q) in screening for eating disorders in community samples. *Behaviour Research and Therapy*.

[29] Polat A, Turan HS, Gündüz N, Alioğlu F, Tural Ü (2019). Predictors of psychosocial functionality in obese women. *Anadolu Psikiyatri Dergisi*.

[30] Karlsson J, Taft C, Rydén A, Sjöström L, Sullivan M (2007). Ten-year trends in health-related quality of life after surgical and conventional treatment for severe obesity: the SOS intervention study. *International Journal of Obesity (2005)*.

[31] Lee YJ, Moon KH, Choi JH, Cho MJ, Shin SH, Heo Y (2013). Validation of the Korean translation of obesity-related problems scale assessing the quality of life in obese Korean. *Journal of the Korean Surgical Society*.

[32] Aguiar PV, Dionisio WÁDS, Souza EADC, Vantini D, Campanholi R, Pinto TCC (2023). Binge eating, depressive symptoms and suicidal ideation in obese candidates for bariatric surgery. *Eating and Weight Disorders: EWD*.

[33] Peterhänsel C, Nagl M, Wagner B, Dietrich A, Kersting A (2017). Predictors of Changes in Health-Related Quality of Life 6 and 12 months After a Bariatric Procedure. *Obesity Surgery*.

[34] Alyahya RA, Alnujaidi MA (2022). Prevalence and Outcomes of Depression After Bariatric Surgery: A Systematic Review and Meta-Analysis. *Cureus*.

[35] Teixeira PJ, Carraça EV, Marques MM, Rutter H, Oppert JM, De Bourdeaudhuij I (2015). Successful behavior change in obesity interventions in adults: a systematic review of self-regulation mediators. *BMC Medicine*.

[36] Mitchell JE, King WC, Courcoulas A, Dakin G, Elder K, Engel S (2015). Eating behavior and eating disorders in adults before bariatric surgery. *The International Journal of Eating Disorders*.

[37] Sockalingam S, Tehrani H, Taube-Schiff M, Van Exan J, Santiago V, Hawa R (2017). The relationship between eating psychopathology and obstructive sleep apnea in bariatric surgery candidates: A retrospective study. *The International Journal of Eating Disorders*.

[38] de Zwaan M, Mitchell JE, Howell LM, Monson N, Swan-Kremeier L, Crosby RD (2003). Characteristics of morbidly obese patients before gastric bypass surgery. *Comprehensive Psychiatry*.

[39] Mento C, Silvestri MC, Muscatello MRA, Rizzo A, Celebre L, Cedro C (2022). The role of body image in obese identity changes post bariatric surgery. *Eating and Weight Disorders: EWD*.

[40] Lo Coco G, Gullo S, Salerno L, Iacoponelli R (2011). The association among interpersonal problems, binge behaviors, and self-esteem, in the assessment of obese individuals. *Comprehensive Psychiatry*.

[41] Hamurcu P, Öner C, Telatar B, Yeşildağ Ş (2015). Obezitenin benlik saygısı ve beden algısı üzerine etkisi. *Türkiye Aile Hekimliği Dergisi*.

[42] Lerdal A, Gay CL, Bonsaksen T, Fagermoen MS (2017). Predictors of physical and mental health in persons with morbid obesity attending a patient education course - a two-year follow-up study. *Health and Quality of Life Outcomes*.

[43] Sarwer DB, Wadden TA, Foster GD (1998). Assessment of body image dissatisfaction in obese women: specificity, severity, and clinical significance. *Journal of Consulting and Clinical Psychology*.

[44] Lee DW, Kim S, Cho DY (2013). Obesity-related quality of life and distorted self-body image in adults. *Applied Research in Quality of Life*.

[45] Sarwer DB, Wadden TA, Moore RH, Eisenberg MH, Raper SE, Williams NN (2010). Changes in quality of life and body image after gastric bypass surgery. *Surgery for Obesity and Related Diseases: Official Journal of the American Society for Bariatric Surgery*.

[46] Bocchieri LE, Meana M, Fisher BL (2002). A review of psychosocial outcomes of surgery for morbid obesity. *Journal of Psychosomatic Research*.

[47] Larsson U, Karlsson J, Sullivan M (2002). Impact of overweight and obesity on health-related quality of life-a Swedish population study. *International Journal of Obesity and Related Metabolic Disorders: Journal of the International Association for the Study of Obesity*.

[48] Martinelli V, Cappa A, Zugnoni M, Cappello S, Masi S, Klersy C (2021). Quality of life and psychopathology in candidates to bariatric surgery: relationship with BMI class. *Eating and Weight Disorders: EWD*.

[49] da Cruz MRR, Branco-Filho AJ, Zaparolli MR, Wagner NF, de Paula Pinto JS, Campos ACL (2018). Predictors of Success in Bariatric Surgery: the Role of BMI and Pre-operative Comorbidities. *Obesity Surgery*.

[50] Sierżantowicz R, Ładny JR, Lewko J (2022). Quality of Life after Bariatric Surgery-A Systematic Review. *International Journal of Environmental Research and Public Health*.

[51] Geller S, Levy S, Hyman O, Jenkins PL, Abu-Abeid S, Goldzweig G (2021). Preoperative body-related emotional distress and culture as predictors of outcomes of bariatric surgery. *Eating and Weight Disorders: EWD*.

[52] Legatto T, Taylor VH, Kidane B, Anvari M, Hensel JM (2022). The Impact of Psychiatric History and Peri-operative Psychological Distress on Weight Loss Outcomes 1 Year After Bariatric Surgery. *Obesity Surgery*.

[53] McCambridge J, Witton J, Elbourne DR (2014). Systematic review of the Hawthorne effect: new concepts are needed to study research participation effects. *Journal of Clinical Epidemiology*.

